# Evaluating the effects of a risk-adapted screening program for familial colorectal cancer in individuals between 25 and 50 years of age: study protocol for the prospective population-based intervention study FARKOR

**DOI:** 10.1186/s12876-020-01247-6

**Published:** 2020-05-05

**Authors:** Sabine Hoffmann, Alexander Crispin, Doris Lindoerfer, Gaby Sroczynski, Uwe Siebert, Ulrich Mansmann, FARKOR Consortium

**Affiliations:** 1 Department for Medical information Processing, Biometry, and Epidemiology, Ludwig-Maximilians University, Munich, D-81377 Germany; 2 Institute of Public Health, Medical Decision Making and HTA, UMIT - Private University for Health Sciences, Medical Informatics and Technology GmbH, Hall in Tirol, A-6060 Austria; 3 German Cancer Consortium (DKTK), Im Neuenheimer Feld 280, Heidelberg, D-69120 Germany

**Keywords:** Colorectal cancer, Screening, Family history, Colonoscopy, Fecal blood test, Shared decision making

## Abstract

**Background:**

Colorectal cancer (CRC) is the second most common malignant disease and the second most common cause of cancer death in Germany. Official CRC screening starts at age 50. As there is evidence that individuals with a family history of CRC have an increased risk of developing CRC before age 50, there are recommendations to start screening for this group earlier. This study aims to evaluate the clinical and economic effects of a risk-adapted screening program for CRC in individuals between 25 and 50 years of age with potentially increased familial CRC risk.

**Methods:**

FARKOR (Familiäres Risiko für das Kolorektale Karzinom) is a population-based prospective intervention study. All members of cooperating statutory health insurance companies between 25 and 50 years of age living in a model region in Germany (federal state of Bavaria, 3.5 million inhabitants in this age group) can participate in the program between October 2018 and March 2020. Recruitment takes place through physicians and through a public campaign. Additionally, insurances contact recently diagnosed CRC patients in order to encourage their relatives to participate in the program. Physicians assess a participant’s familial history of CRC using a short questionnaire. All participants with a family history of CRC are invited to a shared decision making process to decide on further screening options consisting of either undergoing an immunological test for fecal occult blood or colonoscopy. Comprehensive data collection based on self-reported lifestyle information, medical documentation and health administrative databases accompanies the screening program. Longterm benefits, harms and the cost-effectiveness of the risk-adapted CRC screening program will be assessed by decision analytic modeling.

**Discussion:**

The data collected in this study will add important pieces of information that are still missing in the evaluation of the effects and the cost-effectiveness of a risk-adapted CRC screening strategy for individuals under 50 years of age.

**Trial registration:**

German Clinical Trials Register, DRKS-IDDRKS00015097.

## Background

Colorectal cancer (CRC) is the third most common malignant disease worldwide, and the second most common cause of cancer death with many cases occurring in developed countries [1]. In Germany, more than 60,000 individuals are diagnosed with CRC every year and about 25,000 patients with CRC die, making it the second most common cancer and the second most common cause of cancer death in Germany [2].

Most cases of CRC develop slowly from precancerous lesions (adenomas). When adenomas or CRC are detected at an early stage, efficient removal and treatment strategies are available. The possibility to prevent CRC through early adenoma removal is an advantage compared to screening and prevention strategies for most other types of cancer [3–5]. Accordingly, there is growing evidence from randomized controlled trials and observational studies that both CRC incidence and CRC mortality can be reduced through screening followed by preventive interventions [4, 6–8].

Over the past two decades, a general decline in the incidence and mortality of CRC has been observed in many countries. At the same time, an increase in CRC incidence in individuals under 50 years is observed in some countries, in particular in the United States [9] and Australia [10].

In Germany, current screening recommendations for the detection of adenomatous polyps and CRC include biennial testing with an immunological test for fecal occult blood (iFOBT) for those aged 50 years and older and colonoscopy every 10 years starting at age 55. However, about 10% of CRC cases are diagnosed before the age of 55. Moreover, there is evidence that individuals with a family history of CRC have a 2- to 4-fold increased risk of developing colorectal carcinoma [11], resulting in a CRC incidence similar to that of individuals without a family history of CRC who are 10 to 15 years older [12]. Despite recommendations to start CRC screening in this group before 50 years of age, there are currently no risk-adapted screening programs for individuals with a family history of CRC in Germany and in many other countries.

To evaluate a screening program, many aspects have to be considered [13]: the prevalence of the disease and its detection rate in the target population, the risks and burdens implied by the program, the risk of overdiagnosis, as well as aspects of costs. While there are estimates on the prevalence of CRC and of precancerous lesions in individuals with a family history of CRC, these estimates may suffer from an inconsistent treatment of individuals with hereditary forms of CRC [11, 14, 15]. There are estimates of the prevalence of having a family history in different countries and ongoing studies estimating this prevalence in Germany [16]. Finally, there is evidence concerning the usefulness of questionnaires to determine family history of CRC in individuals under 50 years of age [17, 18], but data on the compliance and the frequency of adverse effects of CRC screening in a young screening population are scarce. FARKOR aims to provide the missing information to evaluate the clinical and economic effects of a risk-adapted screening program in individuals between 25 and 50 years of age under real world conditions. This article describes the original protocol for the FARKOR (Familiäres Risiko für das Kolorektale Karzinom) study, a prospective population-based intervention study evaluating the effects of a risk-adapted screening program for familial colorectal cancer in individuals between 25 and 50 years of age in the federal state of Bavaria (Germany). Any modifications of the protocol which may impact on the conduct of the study will require a formal amendment of the protocol which will be communicated to all relevant parties.

### Aims and objectives

The study evaluates the effects of the risk-adapted screening program FARKOR, which aims to identify individuals between 25 and 50 years with a family history of CRC and to provide effective, safe and cost effective screening measures for this risk group. In particular, it aims to answer the following primary research question:

- Does the prevalence of CRC and of advanced adenoma in the selected risk group justify the use of the proposed screening measures?

Moreover, the study will address the following secondary research question:

- Does the introduction of a risk-adapted screening program for individuals between 25 and 50 years lead to a decrease in CRC mortality in this age group?

Finally, secondary research objectives are:

- To assess the complication rate of screening colonoscopies in individuals between 25 and 50 years of age with a family history of CRC
- To evaluate the compliance of a risk-adapted screening program in the target population by estimating participation rates depending on region, patient characteristics and recruitment by medical specialties
- To assess the long-term incremental effectiveness and cost effectiveness of FARKOR based on decision-analytic modeling

## Methods/Design

A prospective, population-based intervention study design was chosen to evaluate the effects of FARKOR. All participants will give written informed consent prior to study entry. Recruitment starts in October 2018 and runs until the end of March 2020. First results are expected to be available in October 2020.

### Participants

The recruitment of participants is multimodal. Participating statutory health insurance (SHI) companies^1^ will inform all patients who have been diagnosed with CRC in the last 18 months that their relatives may have a higher risk of developing CRC compared to the general population. The recently diagnosed CRC-patient can inform his/her relatives about the participation in FARKOR. Moreover, physicians will be able to certify for the FARKOR program by undergoing a specific internet based training program on the identification of individuals with a family history of CRC. As FARKOR physician, they recruit subjects between 25 and 50 years of age to participate in the screening program. Finally, there will be a public campaign for the program as well as a website (https://www.darmkrebs-in-der-familie.de/), which allows interested individuals to choose a close-by FARKOR physician.

Inclusion criteria are: Age between 25 and 50 years, membership of a participating SHI, residence in Bavaria, and written informed consent. Individuals for whom adequate CRC related screening measures already exist are excluded from the study. Exclusion criteria are: Previous diagnosis of CRC, familial adenomatous polyposis or chronic inflammatory bowel diseases (ulcerative colitis, Crohn’s disease) or a known family history of hereditary non-polyposis colorectal carcinoma (HNPCC).

### The FARKOR screening process

Participants will be invited by participating physicians to participate in a short standardized interview regarding their family history, which is based on a simplified version of the Amsterdam and Bethesda criteria [20], and to sign a written informed consent form. Based on this short interview consisting of five simple questions concerning the diagnosis of CRC in first- and second-degree relatives, participants are either classified as potential risk carrier if they indicate having at least one first- or second-degree relative with CRC or as risk free. In a shared decision-making, potential risk carriers and their physicians discuss the risks and benefits of possible CRC screening options, which consist of an iFOBT, a screening colonoscopy, or deferring further CRC screening measures to a later point in life. Patients with a positive iFOBT result will undergo diagnostic workup by colonoscopy and both diagnostic and screening colonoscopies are accompanied by a comprehensive documentation of both macroscopic and histologic findings and of potential complications. In case of pathologic findings, patients will receive treatment according to the established guidelines [21]. Additionally, potential risk carriers will be encouraged to undergo an in-depth interview regarding their family history of CRC in which detailed information concerning age, incidence and mortality of CRC in all first- and second-degree relatives is ascertained.

**Fig. 1:**
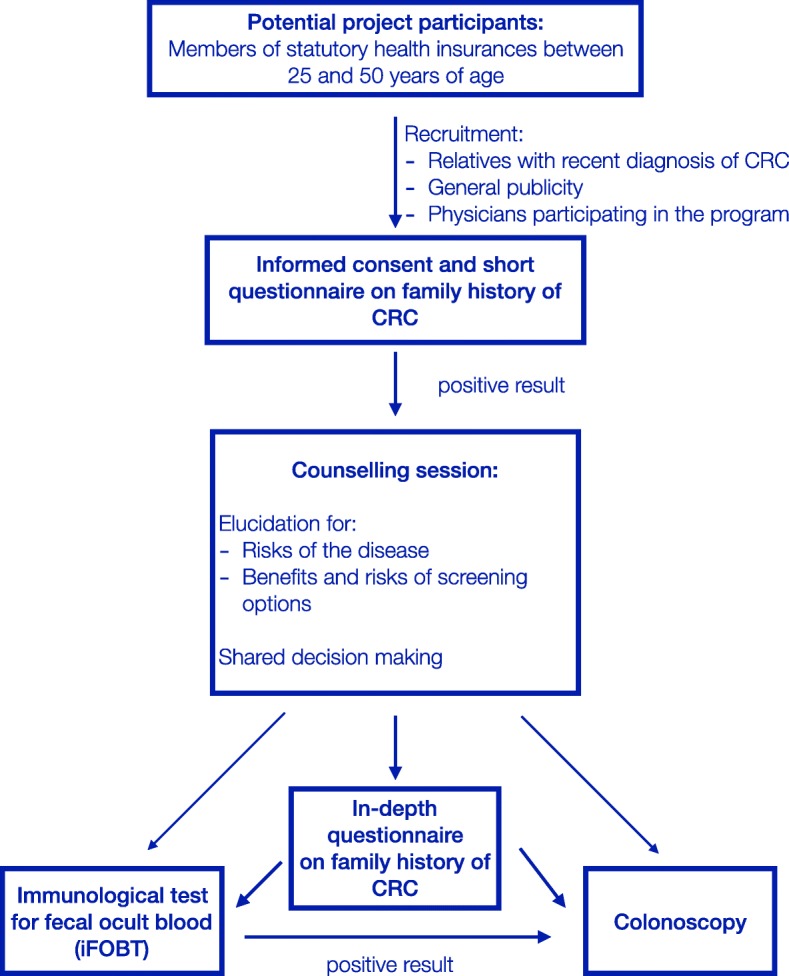
FARKOR screening process

The full FARKOR process is shown in Fig. 1 and consists of the following steps: (1) informed consent, (2) short questionnaire on family history of CRC, (3) shared decision making, (4) offer to complete an in-depth questionnaire on family history of CRC, and (5) further screening measures (iFOBT, colonoscopy, deferral and timing of future screening procedures). All steps in this screening process are accompanied by an internet-based documentation which will be completed by the physicians participating in the program.

Additionally to this FARKOR screening process, all participants (with and without family history of CRC) will be invited to complete an online lifestyle questionnaire based on an instrument previously developed for the RAPS study (Risiko-adaptierte Präventions-Strategien für Darmkrebs) [16]. It consists of questions on pre-existing conditions, participation in prevention programs, lifestyle habits (e.g. smoking, alcohol consumption, nutrition, exercise, drug use…) as well as anthropometric and sociodemographic information (including age, sex, height, weight, and educational level).

### Data collection and data protection

The data collection consists of (1) an internet-based documentation of interviews, results of the shared decision-making, and findings of the proposed screening measures, (2) an internet-based lifestyle questionnaire, which is self-administered by the participants, and (3) health insurance data on the incidence of colorectal adenoma and CRC in Bavaria between the years 2015 and 2020. A central trust center will be performing data linkage and pseudonymization. It will also coordinate all requests from participants regarding transparent information, communication and modalities for the exercise of the rights of the data subject according to the EU General Data Protection Regulation (GDPR) including the right to erasure. The analysis will use anonymized data. The analysis dataset will be stored for 10 years following the guideline of good epidemiological practice (GEP) [22].

### Statistical analysis

#### Primary endpoint

The prevalence of CRC (excluding carcinoma in situ) and of precancerous lesions will be evaluated in participants who underwent a screening colonoscopy. Based on health insurance records, it will also be possible to estimate the incidence of CRC and of precancerous lesions (1) in FARKOR participants with familial CRC risk who decide to defer CRC screening measures to a later point in life (2) in FARKOR participants who are classified as risk free, and (3) in individuals in the target population who do not participate in the FARKOR study.

#### Secondary endpoint

The number of complications arising through screening colonoscopies performed in FARKOR will be considered as secondary endpoint.

#### Comparing the prevalence of CRC and of precancerous lesions in study participants to a general screening population

In order to answer the primary research question, the prevalence of CRC and of precancerous lesions in study participants who undergo colonoscopy will be compared to the findings in a screening population of individuals for whom CRC screening is already implemented in Germany. In particular, the findings concerning the prevalence of CRC and of clinically relevant precancerous lesions (advanced adenoma, defined as adenoma ≥ 10 mm, containing high-grade dysplasia, or villous histology) for colonoscopies performed in FARKOR will be compared to the prevalence observed in screening colonoscopies which are proposed to individuals who are aged 55 years and older in Germany.

#### Modeling long-term outcomes and estimating long-term effects

Due to its short duration of only 18 months (October 2019 until March 2020), the study cannot provide direct evidence concerning the effect of a risk-adapted screening program on CRC mortality in the target population of individuals between 25 and 50 years of age. Therefore, CRC mortality will be estimated indirectly based on information on the stages of detected CRC cases and on the characteristics of precancerous lesions in both FARKOR participants and in individuals in the target population who do not participate in the screening program. A baseline estimate of CRC mortality in the target population for a time period in which no risk-adapted screening was proposed will be estimated based on health insurance data collected in the three years before the start of the FARKOR study.

**Fig. 2:**
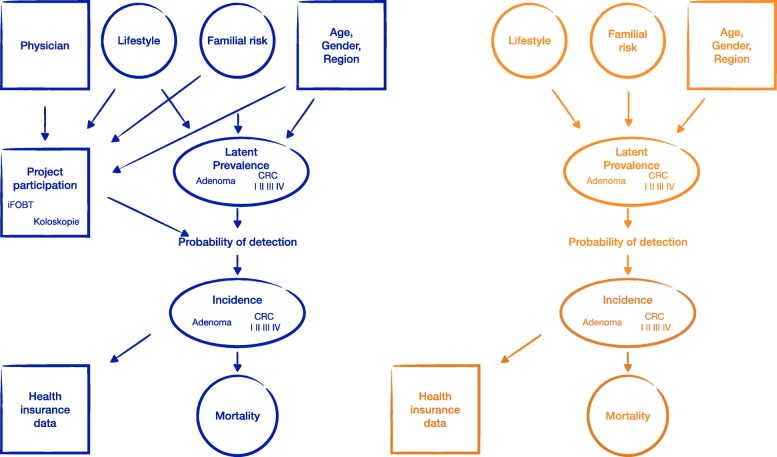
Schematic representation of statistical modeling: The variables for which information is available for the target population (i.e. all SHI members between 25 and under 50 years living in Bavaria) are marked in squares and the variables for which there are uncertainties or missing values for at least part of the target population are marked in circles. The CRC mortality in the study population is shown in blue (left panel) if a risk-adjusted colorectal cancer screening is offered in Bavaria for people with a family history of colorectal cancer between the age of 25 and under 50 years. The CRC mortality in the study population is shown in orange (right panel) if no risk-adjusted colorectal cancer screening is offered for this risk group

A hierarchical model describing the long-term outcomes of the FARKOR program is presented in Fig. 2. The unknown parameters in this hierarchical model can be estimated through Bayesian inference. In order to provide more precise estimates and to reduce some of the uncertainties, it is important to consider not only the direct sources of information (the study database and the health insurance data) but also data from indirect sources of information to make use of all relevant information. Thus, data from the RaPS study [16], the Munich Cancer Registry, the Bavarian Cancer Registry, and the scientific literature will be considered as indirect sources of information.

A detailed description of all statistical methods is provided in a statistical analysis plan, which will be published before the end of data collection.

Finally, additional parameters will be evaluated, including the number of complications due to performed colonoscopies, participation rates as a function of region, patient characteristics and the medical specialties of the recruiting physicians, and the positive predictive value of the iFOBT in individuals between 25 and 50 years of age with a family history of CRC.

### Economic evaluation

The systematic evaluation of long-term health-economic consequences of CRC screening in the population of interest uses a decision-analytic state-transition model [23, 24]. The model reflects the German health care context. It simulates the natural history of colorectal adenoma and cancer development in the target population and assesses the impact of different CRC screening strategies including iFOBT and colonoscopy with diagnostic follow-up and treatment.

The decision model will be applied and calibrated to the German epidemiological setting using the data of the Bavarian Cancer Registry and other German cancer registry data sources. Direct medical costs for screening procedures, diagnostic tests, treatment and follow-up as well as resource utilizations are documented within FARKOR. Outpatient costs will be based on the ’Standardized Rating Scale of the Federal Association of Physicians under the Statutory Health Insurance’ and the ’Statutes of Medical Fees’. Inpatient costs will be derived based on the German Diagnosis Related Groups System.

The economic analyses use a lifelong time horizon. The perspective of the German statutory health insurance (SHI) will be adopted and a 3% annual discount rate will be applied to costs and health outcomes. Outcomes for benefits and harms will include life-years gained (LYG), CRC cases and CRC-related deaths averted, additional complications due to colonoscopy (physical harm) and positive test results (psychological harm). Economic outcomes will include lifetime costs and discounted incremental cost-effectiveness ratios (ICER). Comprehensive sensitivity analyses assess the uncertainty of the results.

The modeling process follows the guidelines of health technology assessment (HTA) and comparative effectiveness research. The analysis will comply with international guidelines and recommendations for decision-analytic modeling, such as the Joint Task Force guidelines of the International Society for Pharmacoeconomics and Outcomes Research (ISPOR) and Society for Medical Decision Making (SMDM) [24, 25], international key principles for HTA, reporting guidelines (CHEERS) and the EUnetHTA guideline for health economic evaluations [26–29].

The decision-analytic state-transition cohort model will be programmed and validated using the decision-analytic software package TreeAge Pro 2017 (TreeAge Software Inc., Williamstown, MA, USA).

### Sample size considerations

**Fig. 3:**
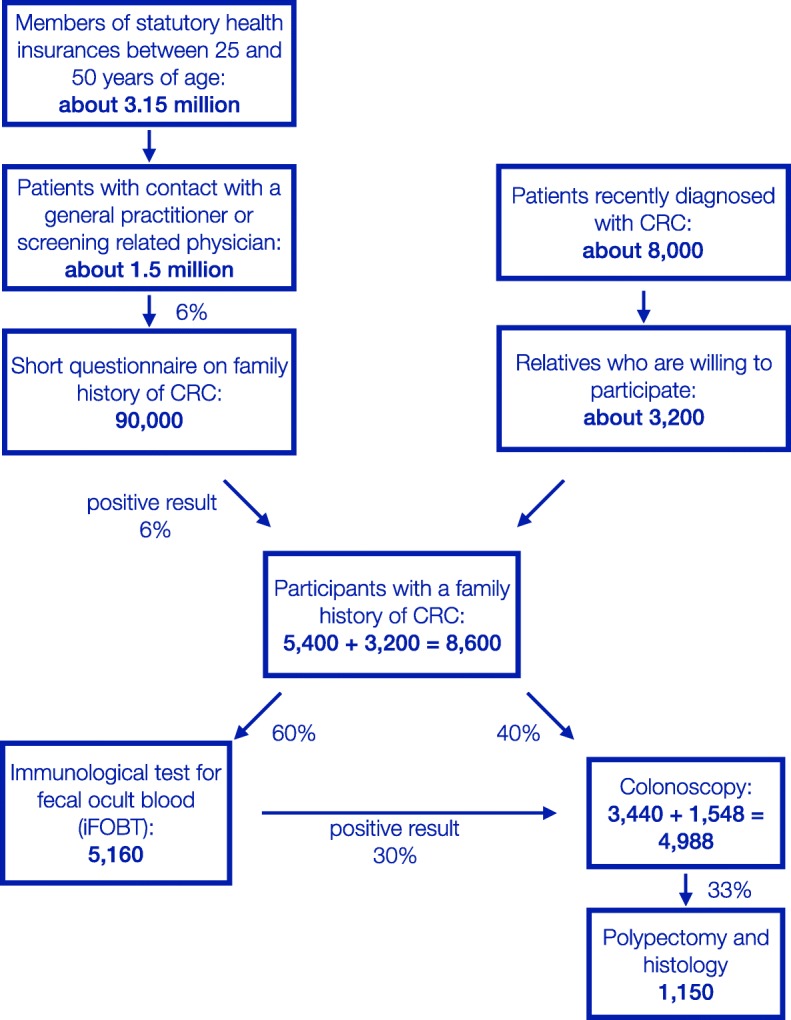
Participant flowchart in the FARKOR program

The statistical analysis will use a population-based data set. In the sample size consideration, it is only possible to use educated guesses for specific parameters (see Fig. 3). The intervention is implemented in a population of about 3.5 million people (Bavarian population between 25 and 50 years of age) of which about 90% (3.15 million people) are SHI members, who represent the target population. Health care statistics show that about 1.5 million individuals in the target population get in contact with a practitioner or a CRC screening related physician every year. We expect that 90,000 (6% of the 1.5 million) will accept to participate in the program and that 6% of these 90,000 will report a positive family history of CRC. This leads to 5,400 potential risk carriers recruited through contact with physicians. Based on the number of CRC cases in Bavaria, it can be estimated that a further 3,200 potential risk carriers will be recruited through their first-degree relatives who are contacted by their SHI. This results in an expected total of 8,600 participants with a family history of CRC. About 40% will decide for a colonoscopy (3,440). The remaining 60% of these subjects will decide for an iFOBT (5,160) of whom 30% will be positive an undergo colonoscopy (1,548). It is expected that 33% of the colonoscopies will produce findings that result in polypectomy and histology (1,150). Given these numbers, we expect to be able to calculate confidence intervals of fair precision for prevalence of CRC or advanced adenoma (±0.01).

## Discussion

The Bavarian population of 13 million represents about 16% of the German population. The target population of this study consists of 3.5 million people. We expect to perform about 5000 colonoscopies after shared decision-making of doctors and study participants who are preselected based on a short questionnaire on family history of CRC. It is of interest to see if the prevalence of CRC and of precancerous lesions corresponds to the findings in a screening population of individuals for whom CRC is already implemented in Germany. About 10% of participants in the Bavarian CRC colonoscopy screening program (regularly starting at the age of 55 years) present with advanced adenoma or CRC [30].

Bavaria has also a full coverage by epidemiologic cancer registries. For the target population the registry reports 2065 CRC cases (ICD10 C18-C21) between 2011 and 2014, corresponding to roughly 500 cases per year. It is of specific interest if the selection process via simple questions about the familial colorectal cancer risk is able to find a relevant part of these affected persons. We assume to recruit 8600 individuals who are classified as potential risk carriers in FARKOR. This number is sufficient to allow precise estimates of the prevalence of CRC and of precancerous lesions and to assess essential aspects of the efficacy of the FARKOR screening approach.

It was not possible to implement a comprehensive and systematic process analysis of the program, which could have addressed the following questions in detail: What are facilitators or inhibitors to perform a familial CRC risk assessment? What are reasons that motivate young adults with established familial risk to refuse further active prevention steps like iFOBT or colonoscopy? However, we will address these questions by collecting data concerning a set of potential facilitators and inhibitors including region, medical specialties, and lifestyle factors.

If FARKOR is able to show that the risk-adapted screening program is effective or even cost-effective, there is the option to make it available in whole Germany and to provide a first risk-adapted approach to a familial CRC screening.

Currently, there is a further CRC screening approach provided in the federal states of Berlin and Brandenburg which is called “Männer, last die Hosen herunter” (“Pants down, men”). This program motivates men between 40 and 50 years of age to participate in early CRC screening. It was shown that men have the same risk patterns regarding advanced adenoma findings about 10 years earlier than women [31]. Furthermore, there is evidence that men are more reluctant in the participation in screening programs and do need specific incentives [32].

## Data Availability

Data sharing is not applicable to this article as it describes a study protocol. The datasets generated and analysed during the current study cannot be shared publicly because participants did not explicitly consent to the sharing of their data as per European Union’s General Data Protection Regulation and the corresponding German privacy laws. Data are available through the Research Ethics Board of the Ludwig-Maximilians-Universität München, Munich/Germany for researchers who meet the criteria for access to confidential data. Please address requests to: ethikkommission@med.uni-muenchen.de.

## Abbreviations

CRC: Colorectal cancer
GEP: Good epidemiological practice
HNPCC: Hereditary non-polyposis colorectal carcinoma
HTA: Health technology assessment
ICER: Incremental cost-effectiveness ratios
ICD10: 10th revision of the International Statistical Classification of Diseases and Related Health Problems
iFOBT: immunological test for fecal occult blood
ISPOR: International Society for Pharmacoeconomics and Outcomes Research
LYG: Life-years gained
SHI: Statutory health insurance
SMDM: Society for medical decision making

## Footnotes

1 Germany has a two-tier healthcare insurance system. Statutory health insurance (SHI) is mandatory for employees with incomes below the contribution assessment ceiling of 56.250 euros per year. The principles of the SHI system are solidarity and self-governance [19]. Contributions to not-for-profit SHI sickness funds are provided mutually by employees and employers. Self-employed citizens and employees with incomes above the contribution assessment ceiling are allowed to opt out of the system in favor of private health insurance companies.
